# The Immunology of Cellular Stress Proteins

**DOI:** 10.3389/fimmu.2013.00153

**Published:** 2013-06-18

**Authors:** Willem Van Eden, Cristina Bonorino, Ruurd Van Der Zee

**Affiliations:** ^1^Infectious Diseases and Immunology, Utrecht UniversityUtrecht, Netherlands; ^2^Instituto Nacional para o Controle do CâncerPorto Alegre, Brazil

Stress proteins or heat shock proteins (HSP) are evolutionary conserved proteins present in every prokaryotic and eukaryotic cell. Their archetypical function is to protect cells and proteins from damage under stressful circumstances. The latter circumstances do include the cell and protein damaging effects of inflammation.

The discovery of mycobacterial HSP60 being a critical antigen in the model of adjuvant arthritis, has led to studies that showed the immuno-dominance of microbial HSP60 and the potential of the microbial HSP induced repertoire of antibodies and T cells to cross-recognize the self-HSP homologs of stressed cells. Since then, the research in the immunology of stress proteins started to comprise a widening spectrum of topics with potential medical relevance. Interestingly, since stress proteins have their activities in both innate and adaptive immunity, they are key elements in the cross-roads between both arms of the immune system.

**Figure 1:**
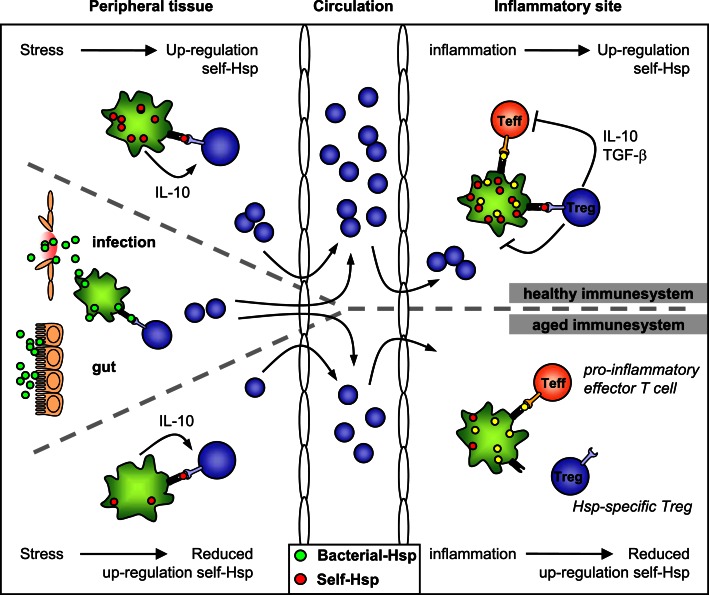
**HSP-specific immunoregulation in the healthy and aged immune system**. Self-HSP-specific T cells reside in the circulation after escape from central tolerance in the thymus. Since HSP are highly conserved, these self-HSP-specific T cells can cross-recognize bacterial HSP. This T cell population can be expanded after exposure to bacterial-HSP at mucosal surfaces like the gut or during infection. At mucosal surfaces, these T cells will be directed toward a regulatory phenotype through mechanisms of mucosal tolerance. In addition, Treg induction and maintenance will be promoted by stress induced HSP expression in peripheral tissues, because up-regulation of self-HSP and presentation of HSP peptides by MHC class II can occur in the absence of co-stimulation. Treg induction will be enhanced by IL-10 produced in response to stress. Furthermore, self-HSP peptides can function as altered peptide ligands for bacterial HSP-specific T cells. During inflammation, HSP will be induced and presented on professional APCs at the inflammatory site, leading to full activation of HSP-specific Treg and local dampening ongoing inflammation. In the aged immune system stress induced HSP expression is decreased. Therefore, reduced HSP inducibility will probably influence both the induction of HSP-specific Treg in the periphery and their activation during inflammation. Ultimately this could result in reduced Treg numbers and function.

Stress proteins or HSP can be considered as functional “biomarkers” of inflammation. They are up-regulated locally during inflammation and interestingly, they seem to function as targets for anti-inflammatory regulatory T cells (Figure 1). In experimental models of autoimmunity, mainly arthritis, administration of HSP peptides has been shown to suppress disease. First clinical trials have shown the anti-inflammatory nature of T cell responses to HSP. In type I diabetes and in rheumatoid arthritis, parenteral and oral administration of HSP peptides were shown to induce a bias in pro-inflammatory T cells, switching them in the direction of regulatory cytokine production (IL4, IL5, and IL-10). In addition a raised level of a marker of T regulatory cells, the transcription factor FoxP3, was noted in the RA trial. Other inflammatory diseases or diseases with inflammatory components which feature the immune imprint of the up-regulated HSP are atherosclerosis, inflammatory bowel diseases, multiple sclerosis, and atopic diseases such as atopic dermatitis and allergic asthma. The review by Borges et al. (2012) discusses the effects of HSP70 on the induction of tolerance at the level of antigen presenting cells and T cells. By this, HSP70 could lead to the development of innovative anti-inflammatory agents to use against autoimmunity and transplant rejection. Shields et al. (2012) have contributed with a review where inhibition and termination of immune responses using BiP (HSP70) are highlighted. They have introduced the term resolution promoting proteins for the aspects of HSPs (Resolution Associated Molecular Patterns or RAMPs). The function of small heat shock proteins (sHSP) in neurological diseases is discussed in the review by Brownell et al. (2012). It highlights the potential of using HSP as novel neuroprotective therapeutics. The ins and outs of HSP as immunoregulatory agents are discussed in more general terms in the review by Coelho and Faria (2012). Aalberse et al. (2012) have broadened the potential of anti-inflammatory effects of HSP in the area of atopic diseases. With the example of HSP60 it is argued that anti-microbial HSP immune reactivity may contribute to atopic disease resistance, suggesting that HSP immunity can constitute the molecular basis of the hygiene hypothesis.

Tumor associated stress proteins seem to qualify as prognostic biomarkers in many tumors. This may be caused by their activity as cellular stress-resistance enhancers. In addition, it may relate to the cell biology of metastasis or to their functions as targets of regulatory T cells. Despite the lack of membrane anchor sequences in HSP, there is ample evidence for cell surface expressed members of stress proteins. In the case of tumors they may then function as targets for NK cells. The review articles of Multhoff et al. (2012) and Calderwood et al. (2012) articulate two additional aspects of stress proteins in cancer development. Whereas both papers discuss the role of HSP as danger molecules, promoting anti-tumor inflammation, Multhoff et al. (2012) also discuss the tumor promoting anti-apoptotic effects of HSP. Calderwood et al. (2012) discuss the anti-tumor immune stimulatory effects of HSP on helper cells and antigen presenting cells. The issue of cell surface expression of HSP seems also relevant for endoplasmic reticulum (ER) stress proteins. As argued in the review by Morito and Nagata (2012), these proteins can also be cell surface expressed and have pathophysiological roles in autoimmunity and inflammation.

A controversy in the area has arisen concerning claims of stress proteins as danger molecules that have the innate quality of inducing inflammatory responses in dendritic cells or other antigen presenting cells. Apart from the possible contribution of contaminating LPS present in earlier recombinant HSP preparations, there is good evidence that cells may perceive stress proteins as danger molecules, indeed. The mechanisms involved in these stress protein activities are ready to be sorted out, amongst others motivated by findings that show additional potential of stress proteins as carriers for protein or oligosaccharide epitopes or as immune stimulatory adjuvants in vaccines. This issue of HSP seen as damage associated molecular patterns (DAMPs) is dealt with in the perspective by Land (2012). The argument in favor of HSP functioning as DAMPs is presented regardless of the final positive or negative regulatory function of HSP. Another aspect of the dual functional role of HSP in immune regulation is highlighted in the mini review by Lee and Repasky (2012). Based on findings that mild hyperthermia can lead to both pro- and anti-inflammatory effects in macrophages, it is proposed that activation state of macrophages is the determining factor in this.
